# Impact of the 2017 AAP clinical guideline on the prevalence of high blood pressure among adolescents in Lagos, Nigeria

**DOI:** 10.3389/fped.2023.1184320

**Published:** 2023-06-23

**Authors:** Ifeoma Atoh, Joseph Ezeogu, Ekanem Ekure, Samuel Ilenre Omokhodion, Fidelis Olisamedua Njokanma

**Affiliations:** ^1^ Department of Paediatrics, College of Medicine, University of Lagos, Lagos, Nigeria; ^2^ Department of Paediatrics, Federal University Teaching Hospital Owerri, Owerri, Nigeria; ^3^ Department of Paediatrics, College of Medicine, University of Ibadan, Ibadan, Nigeria; ^4^ Department of Paediatrics, College of Medicine, Lagos State University, Lagos, Nigeria

**Keywords:** high blood pressure, elevated blood pressure, hypertension, adolescent, secondary school

## Abstract

**Introduction:**

Adolescent high blood pressure (HBP) can lead to several end-organ complications if it continues into adulthood. The 2017 AAP Guideline has lower blood pressure cut-off points and consequently leads to the identification of more people with high blood pressure. This study evaluated the impact of the 2017 American Academy of Pediatrics (AAP) Clinical Guideline on the prevalence of high blood pressure among adolescents when compared to the 2004 Fourth Report.

**Methodology:**

A descriptive cross-sectional study was conducted from August 2020 to December 2020. The selection of 1,490 students, 10–19 years old, was by a two-stage sampling technique. Socio-demographic information and relevant clinical data were obtained using a structured questionnaire. Blood pressure was measured according to standard protocol. Categorical and numerical variables were summarized using frequency, percentages, mean, and standard deviation. The McNemar-Bowker test of symmetry was used to compare the blood pressure values in the 2004 Fourth Report and the 2017 AAP Clinical Guideline. The Kappa statistic was used to test for the degree of agreement between the 2004 Fourth Report and the 2017 AAP Clinical Guideline.

**Results:**

The prevalence rates of high blood pressure, elevated blood pressure, and hypertension among adolescents were 26.7%, 13.8%, and 12.9%, respectively, using the 2017 AAP Clinical Guideline, and 14.5%, 6.1%, and 8.4%, respectively, using the 2004 Fourth Report. The degree of agreement between the 2004 and 2017 guidelines with respect to the classification of blood pressure was 84.8%. The Kappa statistic was 0.71 (CI: 0.67–0.75). The impact of this was a 12.2%, 7.7%, and 4.5% increase in the prevalence of high blood pressure, elevated blood pressure, and hypertension, respectively, using the 2017 AAP Clinical Guideline.

**Conclusion:**

The 2017 AAP Clinical Guideline detects a greater proportion of high blood pressure among adolescents. The adoption of this new guideline in clinical practice and its use in the routine screening of high blood pressure among adolescents is recommended.

## Introduction

The global burden of high blood pressure (HPB) in adolescents is rising and constitutes a major public health challenge, affecting approximately one billion people worldwide and accounting for 9.4 million deaths each year (1). Song et al. (2) revealed a trend of the increasing prevalence of HPB in children and adolescents which was observed during the past two decades with approximately one in seven adolescents aged 12–19 years having elevated BP or hypertension (HTN) during 2013–2016 (3). Risk factors for HBP in adolescents include age, gender, obesity, physical inactivity, family history of HTN in first degree relatives, socioeconomic status, cigarette smoking, and alcohol intake (4). Other risk factors include birth weight, maturity during birth, heredity, renal abnormalities due to diet, coarctation of the aorta, medications, and neoplasm (5). Adolescents with these risk factors need to have their BP evaluated regularly.

However, the evaluation and detection of adolescent HBP can be cumbersome and complex with many challenges and discrepancies (6) given that BP in adolescents is subject to many variables including height, age, and gender which must all be accounted for when attempting to describe “normal” and “abnormal” BP. A solution for this was provided by the advent of evidence-based practice (EBP).

The advent of EBP led to the development of several guidelines which serve to aid physicians in identifying symptoms and signs while making the best clinical decisions based on the most recent available evidence. Amongst these guidelines are the 2004 Fourth Report and the 2017 American Academy of Pediatrics (AAP) Clinical Guideline.

The 2004 Fourth Report included normative data and an adaptation of this data to the childhood growth charts from the Centers for Disease Control and Prevention for the year 2000 (7). Furthermore, overweight or obese children were included in the formation of these tables; the inclusion of these children likely biased normative BP values upwards. However, the increasing prevalence of global childhood obesity (8) and metabolic syndrome (9) implies that the obesity and HTN dyad should not be overlooked. In recognizing this importance, the 2017 AAP Clinical Guideline (10) included an obesity risk assessment tool that aids clinicians in identifying children at risk for obesity, so that guidance on healthy eating and physical activity [at least daily 1 h of physical activity for children (moderate to vigorous)] can be provided early. The 2017 AAP Guideline used new normative BP tables that shifted the 95th percentile down by about 1–4 mmHg, ensuring that adolescent hypertensives are not missed, with easier-to-read tables that have height in inches/centimeters, with values for height and blood pressure percentile all in one.

Given the significant changes in the 2017 AAP Clinical Guideline and its projected impact on the evaluation and management of high blood pressure in adolescents, there was therefore a need for a more systematic evaluation of the impact of its use on the prevalence of HBP in Nigerian children. It is hoped that our findings will provide data for clinicians to implement appropriate changes in their practice to ensure early diagnosis, treatment, and prevention of end-organ damage among adolescents.

## Methodology

### Study design

This was a descriptive cross-sectional study conducted in the Mushin Local Government Area of Lagos State in southwestern Nigeria from August 2020 to December 2020. It is one of the 20 Local Government Areas in Lagos State. Mushin is a densely populated, urban area covering a land area of 14.05 square kilometers with an estimated population of 1,868,743 (11). It shares boundaries in the north with Oshodi-Isolo Local Government, to the east with Somolu and in the south with Surulere. It has one educational district called Educational District VI located in the Oshodi area of Lagos State. There are 129 secondary schools in the Mushin Local Government area comprising 113 private and 16 public secondary schools.

### Study population

This study was conducted among adolescents aged 10–19 years, in 14 (12 private and 2 public) secondary schools in the Mushin Local Government Area of Lagos State. The total population of students in both public and private secondary schools, as obtained from the Educational District VI Oshodi, was 38,266 with 25,125 of them in public secondary schools and 13,141 students in private secondary schools. The ratio of students in public to private schools was approximately 2:1. Our work was done over a 5-month period, August through December 2020. Ethical approval was obtained from the Health Research Ethics Committee of the Lagos University Teaching Hospital numbered as NHREC/DCST/HERC/2659. Approval was also obtained from the Lagos State Ministry of Education with approval number LG/C530/VI/122.

The sample size was determined using the formula for prevalence studies (12).


$$n=Z^{2}p/Qd^{2}$$

where:

*n* = minimum sample size when the study population is >10,000

*z* = Standard normal deviate corresponding to 95% confidence interval = 1.96

*p* = prevalence rate of HBP in adolescents from a previous study done in

Lagos, Nigeria, i.e., 16.5% (0.17) (13).

*q* = (1 − *p*), i.e., 0.83

*d* = precision level was set at 2% (0.02)

Substituting these figures into the formula:

*n* = 1.962 × 0.17 ×  0.830.022 = 1,355.

The minimum sample size was 1,355. An additional 10% (135 pupils) was added to make up for possible non-responses. This brought the total calculated sample size for the study to 1,490. Thus 1,490 adolescents were recruited for the study.

A total of 1,355 students aged 10–19 years who attended private or public secondary school in Mushin Local Government Area of Lagos State whose parents/guardians gave consent; and adolescents aged 18 years or more who gave consent were consecutively enrolled. Adolescents who were known or suspected to have renal conditions, such as acute glomerulonephritis, reno-vascular, and renal parenchymal diseases, and those who were on antihypertensive medications were excluded.

A two-stage sampling technique was used. Based on the student population, a ratio of one public school to six private schools was selected by simple random sampling. Within selected schools, participants were recruited from each class determined by proportional allocation using the school's register. Students were stratified along gender lines (girls and boys) using the class register. Participants were selected from each stratum by simple random sampling (balloting). Before being enrolled in the study, the selected students and their parents gave written and oral consent/assent. Socio-demographic information and relevant clinical data were obtained using a structured questionnaire. Questions were asked to exclude participants based on the presence of symptoms of renal disease and a history of hypertension.

### Procedure

Anthropometry and blood pressure measurements were taken according to standard protocol. Body mass index Z scores were determined using the WHO chart for children 5–19 years (14). Weight was measured to the nearest 0.1 kg with minimal clothing (school uniforms with bare feet) using a standardized weighing scale (SECA model 756). Height was measured with the participant standing straight on bare feet, with both heels placed together, and buttocks, shoulder blades, and head without headgear [in a Frankfort plane (15)] in contact with the measuring rule, and readings recorded to the nearest 0.5 cm using a stadiometer (SECA model 213). Blood pressure was measured using an Accoson sphygmomanometer after the participant had rested in a seated position for 5 min legs uncrossed and flat on the floor. The measurement was done as recommended in the Seventh Report of the Joint National Committee on Prevention, Detection, Evaluation, and Treatment of High Blood Pressure (JNC-7) (16) The cuff covered approximately two-thirds of the arm with the lower border 2 cm above the cubital fossa. The manometer was at the level of the cuff. The radial pulse was palpated, thereafter, the cuff was inflated to record the systolic blood pressure (SBP) by palpation and later deflated. The brachial artery was palpated and its position was noted. The cuff was then inflated to a pressure of 30 mmHg above the level at which the radial pulse was no longer palpable. The stethoscope was placed over the brachial artery in the cubital fossa and the pressure in the cuff was deflated at 2 mmHg per s. The first audible sound (the first Korotkoff sound) was recorded as the SBP. The fifth Korotkoff sound, i.e., the point of disappearance of all sound, was recorded as the diastolic blood pressure (DBP). Blood pressure was measured three times at an interval of 2 min and the mean was recorded. The systolic and diastolic recording, as well as the age, gender, and height in centimeters (cm) of the participant, were used to obtain the blood pressure percentile based on the 2017 AAP Clinical Guideline using the MD CALC (17), a tool developed in partnership with the AAP. The tool is useful for adolescents aged less than 13 years, while the determination of HBP was done manually for adolescents aged 13 years and above. The MSD manual calculator was the tool used in calculating the blood pressure percentiles based on the 2004 Fourth Report (18).

Normal blood pressure (NBP) was defined as an SBP and DBP that are <the 90th percentile for gender, age, and height (19). Elevated BP was defined as an SBP and/or DBP that are either ≥the 90th percentile and <the 95th percentile or between 120/<80 and 129/<80 mmHg (whichever was lower) (10). Stage 1 HTN was defined as an SBP and/or DBP that are either ≥the 95th percentile and <95th percentile +12 mmHg or between 130/80 and 139/89 mmHg (whichever was lower) (10). Stage 2 HTN was defined as an SBP and/or DBP that are either ≥95th percentile +12 mmHg or ≥140/90 mmHg (whichever was lower) (10).

Social class was determined using the socioeconomic indices of the parents as described by Oyedeji (20).

Healthy weight was defined as a Body Mass Index (BMI) for age between the 5th and 85th percentiles, overweight was defined as a BMI for age between the 85th and 95th percentiles, and obesity as a BMI for age above the 95th percentile (21).

### Data analysis

Data were analyzed using the Statistical Package for Social Sciences (SPSS) version 23. Descriptive statistics were used to describe the socio-demographic and anthropometric characteristics. The prevalence rates of HBP, elevated BP, and HTN based on the 2017 AAP Clinical Guideline and 2004 Fourth Report were summarized using frequency and percentages, while numerical variables were summarized using mean and standard deviation. The McNemar-Bowker test of symmetry was used to compare the difference in the prevalence of HBP values using the 2017 AAP Clinical Guideline and the 2004 Fourth Report. The Kappa statistic was used to test for the degree of agreement between the 2004 Fourth Report and the 2017 AAP Clinical Guideline.

## Results

### Characteristics of the study participants

Of the initial 1,500 adolescents invited into the study, 1490 completed responses, giving a response rate of 99.3%. There was approximately equal representation of boys and girls and the mean age of the participants was 14.39 ± 2.79 years. The highest proportion of participants (649: 43.6%) belonged to the upper socio-economic class. The majority of the participants (83.5%) had a healthy weight as assessed by BMI, while 88 (5.9%) were underweight. There were 132 (8.9%) overweight and 25 (1.7%) obese participants (Table 1).

**Table 1:** Distribution of characteristics of study participants by age.

Variable	Frequency (*n* = 1,490)	Percentage
Age (years)		
10–13	590	39.6
14–16	504	33.8
17–19	396	26.6
Mean age ± SD	14.39 ± 2.79	
Sex		
Male	744	49.9
Female	746	50.1
Socioeconomic status		
Upper class (I and II)	649	43.6
Middle class (III)	545	36.6
Lower class (IV and V)	296	19.8
Body mass index (BMI *Z* score)		
Underweight (<−2SD)	88	5.9
Healthy weight (≥−2SD–≤+1SD)	1,245	83
Overweight (>+1 SD–≤+2 SD)	1 32	8.9
Obese (>+2 SD)	25	1.7

BMI, body mass index, SD, standard deviation.

### Prevalence of HBP among participants based on the 2004 Fourth Report

The overall prevalence of HBP in the study population based on the 2004 Fourth Report was 14.5% (*n* = 216), while 1,274 (85.5%) had NBP.

### Prevalence of HBP among participants based on the 2017 AAP clinical guideline

Based on the 2017 AAP Guideline, 398 participants had HBP with a prevalence rate of 26.7%.

### Pattern of HBP among participants based on the 2004 Fourth Report

Table 2 shows the pattern of HBP when based on the 2004 Fourth Report. The prevalence rate of prehypertension was 6.1% while the prevalence rate of stage 1 HTN was 4.8% and the prevalence rate of stage 2 HTN was 3.6%. With respect to the 216 participants that had HBP, 42.6% had prehypertension, 32.9% had stage 1 HTN, and 24.5% had stage 2 HTN. The mean SBP and DBP increased steadily across the various blood pressure categories.

**Table 2:** Pattern of high blood pressure among participants based on the 2004 Fourth Report.

Variable	Frequency	Percentage of the study population	Percentage of high blood pressure	SBP (mean ± SD)	DBP (mean ± SD)
Prehypertension	92	6.1	42.6	127.95 ± 4.34	72.82 ± 7.74
Stage 1 HTN	71	4.8	32.9	133.21 ± 3.22	81.03 ± 7.48
Stage 2 HTN	53	3.6	24.5	140.58 ± 8.89	94.00 ± 4.65
High BP	216	14.5	100	132.78 ± 7.45	81.99 ± 7.08

SBP, systolic blood pressure; DBP, diastolic blood pressure; HTN, hypertension; SD, standard deviation; CI, confidence interval.

### Pattern of HBP among participants based on the 2017 AAP clinical guideline

Table 3 shows the pattern of HBP. The prevalence rate of elevated BP was 13.8%, the prevalence rate of stage 1 HTN was 9.3%, and the prevalence rate of stage 2 HTN was 3.6%. Among the 398 participants who had HBP, slightly more than half (51.5%) had elevated BP. The mean SBP was 123.10 ± 3.62 for participants with elevated BP, 132.16 ± 3.27 for participants with stage 1 HTN, and 140.61 ± 8.81 for participants with stage 2 HTN. The mean DBP also showed a steady increase and was highest (93.98 ± 4.52) for participants with stage 2 HTN. The prevalence of HTN among the boys was higher than the girls based on the 2017 AAP Clinical Guideline at 28.5% (212). This same effect was seen both in prehypertension/elevated BP and stage 1 HTN but not stage 2 HTN. The same pattern was observed among the girls. This is shown in Table 3.

**Table 3:** Pattern of high blood pressure among participants based on the 2017 AAP guideline.

Variable	Frequency	Percentage of the study population	Percentage of high blood pressure	SBP (mean ± SD)	DBP (mean ± SD)
Elevated	205	13.8	51.5	123.10 ± 3.62	68.45 ± 6.83
Stage 1 HTN	139	9.3	34.9	132.16 ± 3.27	80.98 ± 6.46
Stage 2 HTN	54	3.6	13.6	140.61 ± 8.81	93.98 ± 4.52
High BP	398	26.7	100	128.64 ± 7.78	76.29 ± 8.19

AAP, American academy of pediatrics; SBP, systolic blood pressure; DBP, diastolic blood pressure; HTN, hypertension; SD, standard deviation; CI, confidence interval.

### Comparison of prevalence and patterns of HBP based on the 2004 Fourth Report and the 2017 AAP clinical guideline

Overall, the prevalence of HBP was higher based on the 2017 AAP Clinical Guideline than on the 2004 Fourth report (26.7% vs. 14.5%) with an increase of 12.2% (*p* = 0.001). The increase was reflected in the prehypertension and stage 1 HTN groups (*p* = 0.001 each) but not in the stage 2 HTN group (*p* = 0.999). This is shown in Table 4.

**Table 4:** Comparison of prevalence and pattern of high blood pressure using the 2004 fourth report and 2017 AAP clinical guideline.

Variable	2004 Fourth Report *n* (%)	2017 AAP guideline *n* (%)	Difference in prevalence % (95% CI)	*p*-Value
PreHTN/elevated	92 (6.2)	205 (13.8)	7.6 (5.7, 9.5	0.001*
Stage 1 HTN	71 (4.8)	139 (9.3)	4.5 (3.4, 5.7)	0.001*
Stage 2 HTN	53 (3.5)	54 (3.6)	0.1 (0.08, 013)	0.999
Overall high BP	216 (14.5)	398 (26.7)	12.2 (10.5, 13.9)	0.001*

All *p*-values were calculated using the McNemar-Bowker test of symmetry. HTN, hypertension; BP, blood pressure; CI, confidence interval.

* Significant.

### Comparison of BP patterns according to the age group of participants based on the 2004 Fourth Report and the 2017 AAP clinical guideline

Within each age group, the prevalence of HBP was significantly higher based on the 2017 AAP Clinical Guideline (*p* = 0.001). The higher prevalence affected prehypertension/elevated BP and stage 1 HTN but not stage 2 HTN. This is shown in Table 5.

**Table 5:** Comparison of blood pressure patterns according to the age groups of participants using the 2004 fourth report and 2017 AAP guideline.

Variable	Blood pressure	2004 Fourth Report	2017 AAP guideline	*p*-Value
Age group		*n* (%)	*n* (%)	
**10–13 (***n* = 590)				
	Normal BP	504 (85.4)	466 (79.0)	0.001*
	High BP	86 (14.6)	124 (21.0)	0.001*
	**High BP**			
	Pre HTN/elevated BP	27 (4.6)	42 (7.1)	0.035*
	Stage 1 HTN	30 (5.1)	52 (8.8)	0.001*
	Stage 2 HTN	29 (4.9)	30 (5.1)	0.999
**14–16** (*n* = 504)				
	Normal BP	425 (84.3)	367 (72.8)	0.001*
	High BP	79 (15.7)	137 (27.2)	0.001*
	**High BP**			
	Pre HTN/elevated BP	38 (7.5)	82 (16.3)	0.001*
	Stage 1 HTN	27 (5.4)	41 (8.1)	0.001*
	Stage 2 HTN	14 (2.8)	14 (2.8)	1.000
**17–19** (*n* = 396)				
	Normal BP	345 (87.1)	259 (65.4)	0.001*
	High BP	51 (12.9)	137 (34.6)	0.001*
	**High BP**			
	Pre HTN/elevated BP	27 (6.8)	81 (20.5)	0.001*
	Stage 1 HTN	14 (3.5)	46 (11.6)	0.001*
	Stage 2 HTN	10 (2.5)	10 (2.5)	0.999

All *p*-values were calculated using the McNemar-Bowker test of symmetry. BP, blood pressure; HT, hypertension.

* Significant.

### Level of agreement between the 2004 Fourth Report and the 2017 AAP clinical guideline on the classification of blood pressure

**Figure 1:**
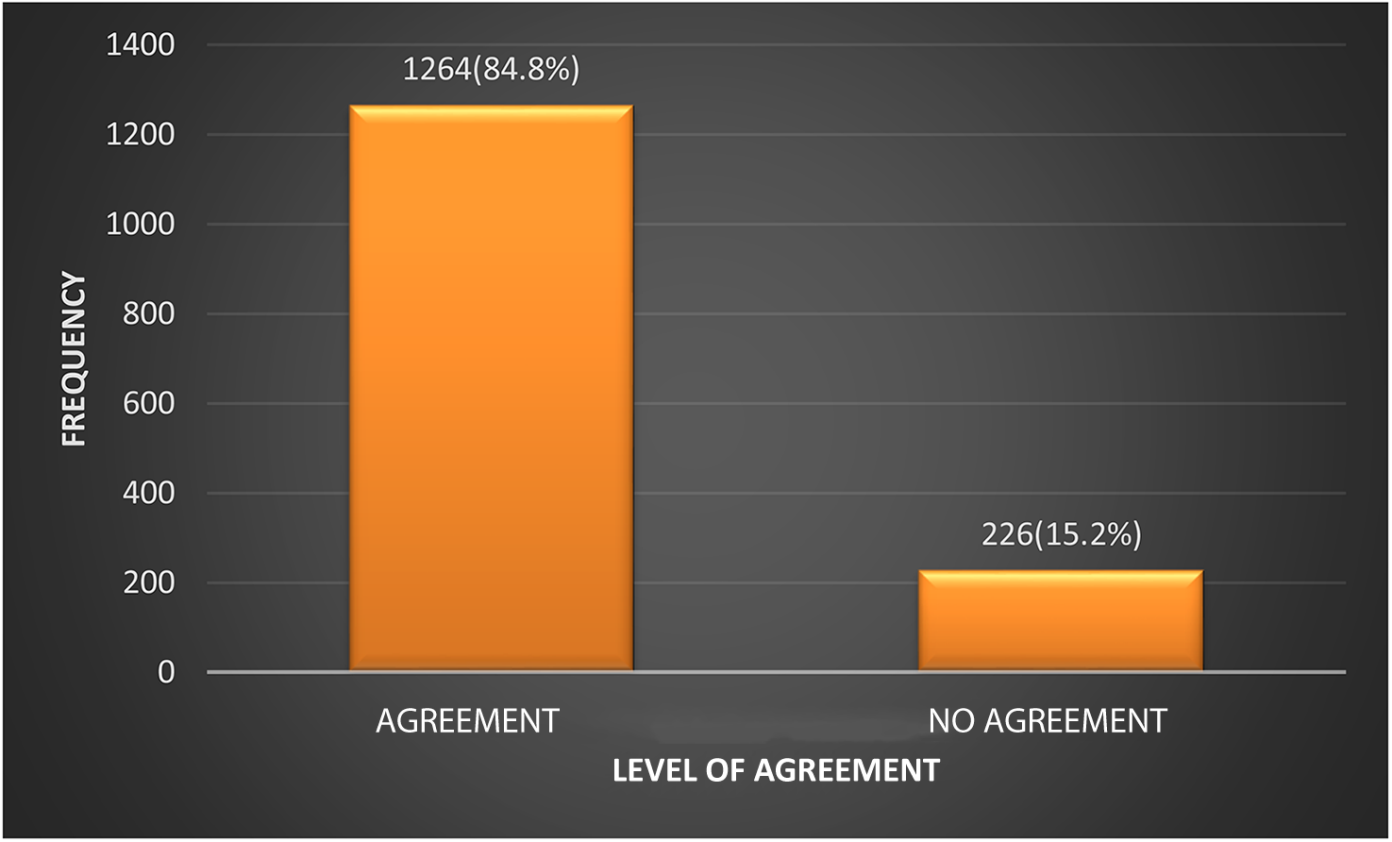
Level of agreement between the 2004 Fourth Report and the 2017 AAP clinical guideline on the classification of BP.

The degree of agreement between the 2004 and the 2017 guidelines on the classification of the BP of participants was assessed. Figure 1 shows that in 1,264 (84.8%) cases the two sets of guidelines agreed on classifying the participants as normal or abnormal. In 226 (15.2%) cases there was no agreement between the 2004 and 2017 guidelines. The Kappa statistic was 0.71 (CI: 0.67–0.75).

## Discussion

The current study demonstrates that there is a high burden of HBP amongst adolescents in Mushin, Lagos, Nigeria, a low-income country. Our study demonstrates that the 2017 AAP Clinical Guideline commonly identifies more adolescents as hypertensives than the 2004 Fourth Report.

It confirms that pre-hypertension and elevated BP are common using either the 2004 Fourth Report or the 2017 AAP Clinical Guideline. Within each age group, the prevalence of HBP was significantly higher when based on the 2017 AAP Clinical Guideline. This accentuates the sensitivity of the 2017 AAP Clinical Guideline in the detection of HBP amongst adolescents.

The first significant finding of this study is the establishment of the fact that the prevalence of HBP amongst adolescents in the current study was 14.5% using the 2004 Fourth Report and 26.7% with the 2017 AAP Clinical Guideline. Some previous studies had demonstrated a higher prevalence of HBP among adolescents of 27.5% (13) and 22. 5% (22) using the 2004 Fourth Report. This disparity may be attributable to the remarkably lower prevalence of overweight participants in the current study, 1.7%, compared to 9.6% and 12.6% in the previous Lagos and Enugu studies (13, 22). Using the 2017 AAP Guideline, the prevalence of HBP found in the current study was 26.7%. This difference in prevalence was significant and could be a result of the fact that the new reference tables in the 2017 AAP Clinical Guideline were produced excluding data from overweight and obese children. If this is so, the lower normative BP values in the 2017 AAP Clinical Guideline imply that there would be an increase in the prevalence of HBP in adolescents and a strong association between overweight/obesity and blood pressure. This exclusion resulted in 1–4 mm Hg drops in the elevated (≥90th percentile) and HTN (≥95th percentile) BP thresholds for children (23).

As anticipated, applying the 2017 AAP Clinical Guideline resulted in an increase in the prevalence rates of elevated BP (7.7%) and HTN (4.5%) over what they might have been using the 2004 Fourth Report. This is akin to the findings by Bell et al. (24), Sharma et al. (25), and Khoury et al. (26) in the US among children and adolescents. The implication of this is that the 2017 AAP Clinical Guideline is a more sensitive tool for the detection of HBP and fewer children with HBP will be missed. Missing HBP in any child exposes that child to adult HTN and hypertensive target organ damage including cerebrovascular accidents, retinopathy, coronary heart disease/myocardial infarction and heart failure, proteinuria and renal failure, and atherosclerotic changes (27).

Another striking finding with respect to age in the present study was that the prevalence of HBP increased progressively with the age group when the 2017 AAP Clinical Guideline was applied. This differed markedly from the pattern when using the 2004 Fourth Report in which the prevalence was lowest in the oldest age group and rates did not differ as much across age groups. This finding with the 2004 Fourth Report was unusual because BP is known to progressively increase with age.

The degree of agreement between the 2004 and 2017 guidelines with respect to the classification of BP was reasonably high at 84.8%, corroborating an earlier report of 84.8% by Sharma et al. (25) The corollary observation was that the 2004 and 2017 guidelines were at variance in the classification of 15.2% of participants. In all cases, as seen in the present study, affected participants were reclassified from normal to high blood pressure or from prehypertension to hypertension. There was no downward reclassification (23, 24, 28).

### Strengths and limitations of the study

The current report is one of the earliest in Nigeria that used the 2017 AAP Clinical Guideline and the first to prospectively compare with the 2004 Fourth Report recommendation, with a fairly large sample of participants covering the full range of adolescent years. Evidence has been provided showing high prevalence rates of elevated BP and HTN among adolescents in secondary schools in Lagos. Due to limited resources, the study was done only in adolescents in secondary students to the exclusion of those who were not in school. It is plausible that social factors that influence BP may differ between the two subgroups of adolescents.

## Conclusion

That more than a quarter of the adolescents in the present study had high blood pressure is indicative of a high prevalence, underscoring the need for routine blood pressure screening. Application of the 2017 AAP Clinical Guideline instead of the 2004 Fourth Report results in a substantial increase in the prevalence of high blood pressure among adolescents in secondary schools.

## Data Availability

The original contributions presented in the study are included in the article, further inquiries can be directed to the corresponding author.
